# Comprehensive analysis of molecular mechanisms underlying kidney stones: gene expression profiles and potential diagnostic markers

**DOI:** 10.3389/fgene.2024.1440774

**Published:** 2024-11-13

**Authors:** Kaisaier Aji, Aierken Aikebaier, Asimujiang Abula, Guang Lu Song

**Affiliations:** ^1^ Urology Department, The First Affiliated Hospital of Xinjiang Medical University, Urumqi, China; ^2^ Department of Imaging Center, The First Affiliated Hospital of Xinjiang Medical University, Urumqi, China

**Keywords:** kidney stones, molecular mechanism, gene expression profiles, machine learning, gene expression

## Abstract

**Background:**

The study aimed to investigate the molecular mechanisms underlying kidney stones by analyzing gene expression profiles. They focused on identifying differentially expressed genes (DEGs), performing gene set enrichment analysis (GSEA), weighted gene co-expression network analysis (WGCNA), functional enrichment analysis, and screening optimal feature genes using various machine learning algorithms.

**Methods:**

Data from the GSE73680 dataset, comprising normal renal papillary tissues and Randall’s Plaque (RP) tissues, were downloaded from the GEO database. DEGs were identified using the limma R package, followed by GSEA and WGCNA to explore functional modules. Functional enrichment analysis was conducted using KEGG and Disease Ontology. Various machine learning algorithms were used for screening the most suitable feature genes, which were then assessed for their expression and diagnostic significance through Wilcoxon rank-sum tests and ROC curves. GSEA and correlation analysis were performed on optimal feature genes, and immune cell infiltration was assessed using the CIBERSORT algorithm.

**Results:**

412 DEGs were identified, with 194 downregulated and 218 upregulated genes in kidney stone samples. GSEA revealed enriched pathways related to metabolic processes, immune response, and disease states. WGCNA identified modules correlated with kidney stones, particularly the yellow module. Functional enrichment analysis highlighted pathways involved in metabolism, immune response, and disease pathology. Through machine learning algorithms, KLK1 and MMP10 were identified as optimal feature genes, significantly upregulated in kidney stone samples, with high diagnostic value. GSEA further elucidated their biological functions and pathway associations.

**Conclusion:**

The study comprehensively analyzed gene expression profiles to uncover molecular mechanisms underlying kidney stones. KLK1 and MMP10 were identified as potential diagnostic markers and key players in kidney stone progression. Functional enrichment analysis provided insights into their roles in metabolic processes, immune response, and disease pathology. These results contribute significantly to a better understanding of kidney stone pathogenesis and may inform future diagnostic and therapeutic strategies.

## 1 Introduction

In the past 30 years, the incidence of kidney stones has significantly increased due to environmental changes, including imbalanced diet and restricted physical activity (Wigner et al., 2021). During this period, the incidence of kidney stones has nearly doubled, with a particularly notable increase among adolescents and young women (Hill et al., 2022; Tasian et al., 2016). Kidney stones, as a common disease of the urinary system, with calcium oxalate (CaOx) stones accounting for the highest proportion, approximately 75%–85% of all urinary stones (Sakhaee et al., 2012; Thongprayoon et al., 2020). The adherence of calcium oxalate (CaOx) crystals to renal tubular epithelial cells serves as a critical contributor in the development of CaOx stone formation (Qi et al., 2020). To further explain this phenomenon, we introduced the concept of Randall’s Plaque (RP). This is a widely accepted theory aimed at explaining the formation mechanism of calcium oxalate stones (Khan et al., 2021). Randall’s plaque was initially described by Randall in 1937 as a pre-stone lesion of kidney stones, and the vast majority of kidney stones belong to the calcium oxalate stone type (Randall, 1937).

In order to further explore the biological characteristics and formation mechanism of kidney stones, we first obtained gene expression profile datasets covering normal renal papillary tissue and Randall’s Plaque (RP) tissue from the GEO database. Subsequently, we rigorously screened differentially expressed genes (DEGs) using the limma algorithm and visualized these differences through volcano plots and heatmaps. To further analyze the functional characteristics and key functional modules of these differentially expressed genes, we utilized the ClusterProfiler and WGCNA tools. Through these analyses, we not only understood the role of these genes in the formation of kidney stones but also identified potential key regulatory pathways. Through the combined application of various machine learning algorithms, we successfully screened out the optimal feature genes KLK1 and MMP10. To gain deeper insights into the roles of these two genes in kidney stones, we compared their expression levels and explored their biological significance. In addition, we used GSEA and CIBERSORT algorithms to further evaluate the relevance of these two optimal feature genes to the formation of kidney stones and their impact on immune cell infiltration. These analyses provide us with a deeper understanding of the biological characteristics and potential mechanisms of kidney stones, and offer new directions for future research.

## 2 Methods

### 2.1 Data collection and processing

We downloaded the GSE73680 dataset from the GEO database, which includes gene expression profiles of 33 normal renal papillary tissues and 29 Randall’s Plaque (RP) tissues.

### 2.2 Identification of differentially expressed genes (DEGs) and gene set enrichment analysis

Differentially expressed genes (DEGs) between Randall’s Plaque (RP) tissues and normal renal papillary tissues were discerned utilizing the “limma” R package. During the screening process, we applied strict criteria of |log2 FC| > 0.5 and *p*-value <0.05. To visually display these differential genes, volcano plots and heatmaps were generated using R software version 4.3.0. Subsequently, to explore the functional characteristics of these differential genes, gene set enrichment analysis (GSEA) was performed using the “ClusterProfiler” R package (Wu et al., 2021). Enrichment significance was determined at a set q value (False Discovery Rate, FDR) < 0.05 and p. adjust <0.05, with normalized enrichment scores (NES) serving as assessment metrics. A positive NES signifies upregulation, while a negative value indicates downregulation.

### 2.3 Weighted gene co-expression network analysis (WGCNA)

Weighted gene co-expression network analysis was performed utilizing the “WGCNA” R package to detect essential functional modules that could elucidate the biological traits of kidney stone samples (Langfelder and Horvath, 2008). Before constructing the network, we carefully examined the merged gene matrix to ensure no abnormal samples were erroneously included, thus ensuring the accuracy of subsequent sample clustering. Genes displaying comparable expression profiles were grouped into coherent co-expression modules following the analysis of the weighted correlation adjacency matrix and cluster analysis. Subsequently, the topological overlap matrix (TOM) was constructed from the adjacency matrix to partition genes into modules based on dissimilarities. A cutting height of 0.25, a minimum module size of 50, and a soft threshold power of 24 (achieving a scale-free R^2^ of 0.9) were chosen during this process. Following module partitioning, gene significance (GS) and module membership (MM) values were computed for each gene. To explore the relationship between functional modules and kidney stones, Spearman correlation coefficients and corresponding *p* values were computed between control groups, kidney stone groups, and various functional modules. Finally, key modules most relevant to kidney stones were determined, and core genes were extracted for further analysis.

### 2.4 Functional enrichment analysis

Following the analysis, we identified overlapping candidate genes from differentially expressed genes (DEGs) and module genes. A Venn diagram was generated using appropriate software to visually represent the gene overlaps. Enrichment analysis of the Kyoto Encyclopedia of Genes and Genomes (KEGG) and Disease Ontology (DO) was performed using the “clusterProfiler” and “DOSE” R packages to explore the functions and pathways linked to the common candidate genes (Wu et al., 2021).

### 2.5 Screening of optimal feature genes

A variety of machine learning algorithms, including LASSO, SVM-REF, RF, Boruta, XGBOOST, GBM, and decision trees, were employed for disease status prediction and identification of key prognostic factors. Specifically, LASSO regression was utilized to select and regularize variables to improve the predictive accuracy of the model (Sepulveda, 2020). Additionally, we employed widely-used supervised learning methods such as SVM and RF to prevent overfitting and produce interpretable results (Kanehisa and Goto, 2000; Yu et al., 2015). Furthermore, we used the SVM-RFE algorithm to precisely locate the most discriminative gene set, laying the foundation for determining the most appropriate feature genes (Li et al., 2020). In the context of predictive problems, decision trees, as the cornerstone of random forests (RF), play an important role, and we determined the optimal number of trees through testing. Following the construction of the Random Forest (RF) model, the backward elimination technique was applied to identify significant genes, with a gene importance threshold of greater than 2 being a pivotal selection criterion in the RF algorithm (Tian et al., 2020). Additionally, the Boruta algorithm significantly influenced the feature selection process by creating shadow features and comparing Z-scores of real features with shadow features (Degenhardt et al., 2019). XGBOOST (XGB), similar to random forests, also originates from decision tree-based ensemble learning (Bentéjac et al., 2021). However, XGB further improves the predictive ability and robustness of the model through its unique sequential learning strategy and embedded gradient descent algorithm (Chen and Guestrin, 2016; Shwartz-Ziv and Armon, 2022). Finally, decision trees excel in handling nonlinear relationships between variables and provide excellent data visualization (Quinlan, 1986). Through the comprehensive application of these machine learning algorithms, we successfully determined the optimal feature genes.

### 2.6 Expression and diagnostic significance of optimal feature genes

Wilcoxon rank-sum tests were performed to evaluate the expression levels of optimal feature genes in kidney stone samples compared to control samples. Additionally, to further validate the predictive efficacy of these feature genes, receiver operating characteristic (ROC) curves were used for evaluation.

### 2.7 GSEA and correlation analysis of optimal feature genes

Moreover, the biological significance of optimal feature genes was explored using gene set enrichment analysis (GSEA) with the “c2. cp.kegg.v11.0. symbols” gene set as a reference. To ensure result accuracy, gene set permutation was iterated 1,000 times for each analysis to derive normalized enrichment scores. Enrichment of gene sets with q values below 0.05 was deemed significant. Furthermore, Pearson correlation analysis was performed to evaluate the relationships between expression levels of optimal feature genes and identify correlations among them.

### 2.8 Evaluation of marker gene sets and immune cell infiltration

The CIBERSORT algorithm, as a gene expression-based deconvolution method, was used to assess the variation of the internal genome of samples and compare it with the changes in other genes (Newman et al., 2019)In this study, the CIBERSORT algorithm was employed to ascertain the infiltration status of 22 immune cells in both normal and kidney stone samples. Boxplots were utilized for a visual representation of the variances in immune cell composition among patients exhibiting diverse immune patterns (Leek et al., 2012). Through Wilcoxon rank-sum tests, we conducted differential analysis of the proportions of these immune cells, considering *p* values less than 0.05 as statistically significant differences.

Moreover, to assess the relative levels of 50 marker gene sets (h.all.v7.5.1. symbols.gmt) in the combined dataset, the single-sample gene set enrichment analysis (ssGSEA) algorithm was utilized. Additionally, Spearman correlations were computed to investigate the relationship between these marker gene sets and the optimal feature genes.

## 3 Results

### 3.1 Identification of DEGs and gene set enrichment analysis in the kidney stone dataset

**FIGURE 1:**
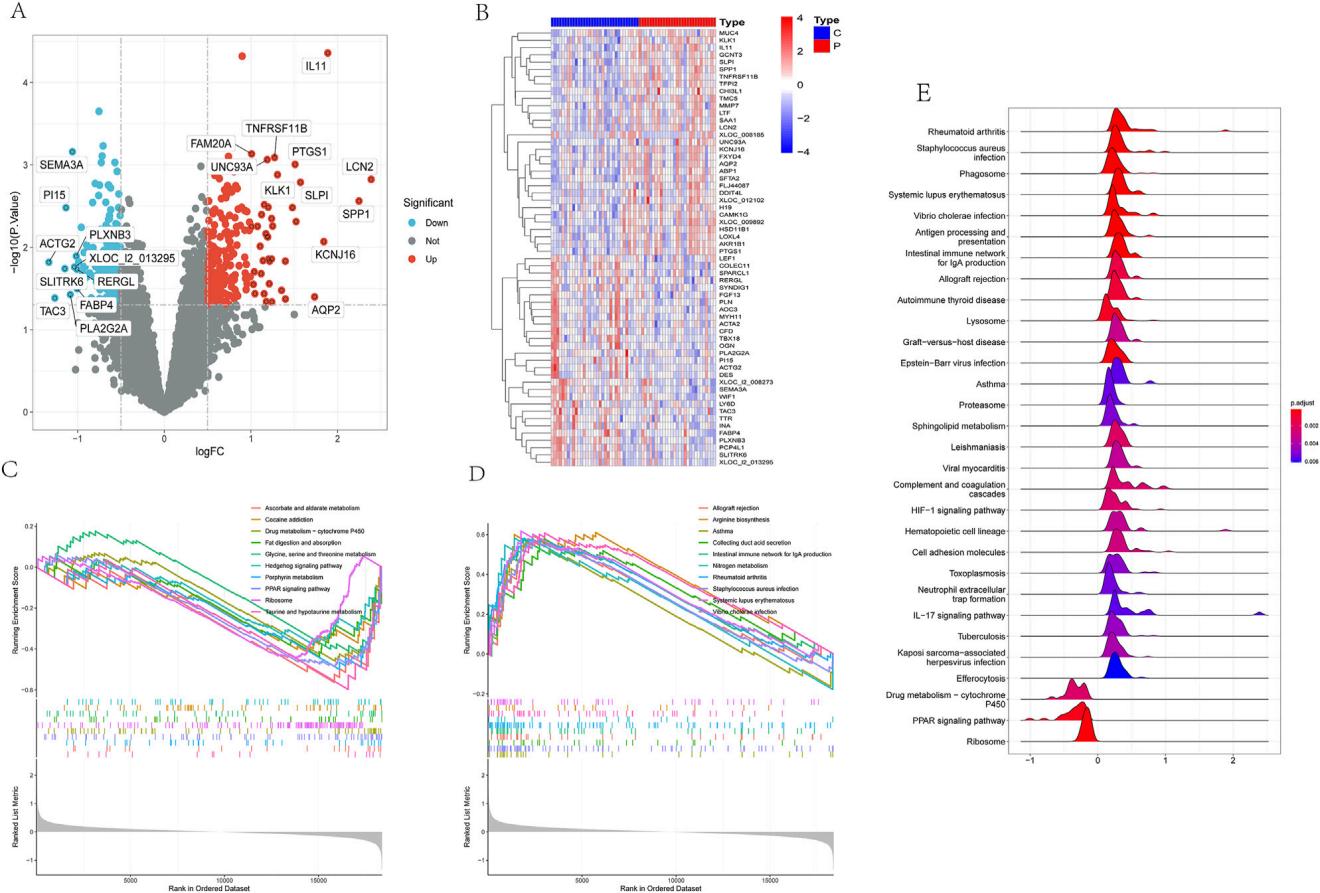
Identification of DEGs and gene set enrichment analysis in the kidney stone dataset.

Following a thorough analysis, a detailed list of 412 Differentially Expressed Genes (DEGs) was compiled. Among these DEGs, 194 genes were observed to be downregulated while 218 genes were upregulated in the disease state, as illustrated in volcano plots and heatmaps (Figure 1A). Additionally, a heatmap focusing on the top 60 genes exhibiting the highest variance was generated to investigate prominent differences between the disease and control groups (Figure 1B). Furthermore, to deepen the analysis, Gene Set Enrichment Analysis (GSEA) was utilized to explore the dataset.

Based on the Normalized Enrichment Score (NES), we categorized enriched pathways into downregulated and upregulated groups. In the downregulated group, several significantly enriched pathways included taurine and hypotaurine metabolism, ascorbate and aldarate metabolism, porphyrin metabolism, PPAR signaling pathway, drug metabolism-cytochrome P450, ribosome, fat digestion and absorption, glycine, serine and threonine metabolism, and cocaine addiction, among others (Figure 1C). In the upregulated group, significantly enriched pathways included systemic lupus erythematosus, arginine biosynthesis, *Vibrio cholerae* infection, nitrogen metabolism, rheumatoid arthritis, intestinal IgA production immune network, collecting duct acid secretion, *Staphylococcus aureus* infection, and asthma, among others (Figure 1D).

### 3.2 Selection of WGCNA modules and hub genes

**FIGURE 2:**
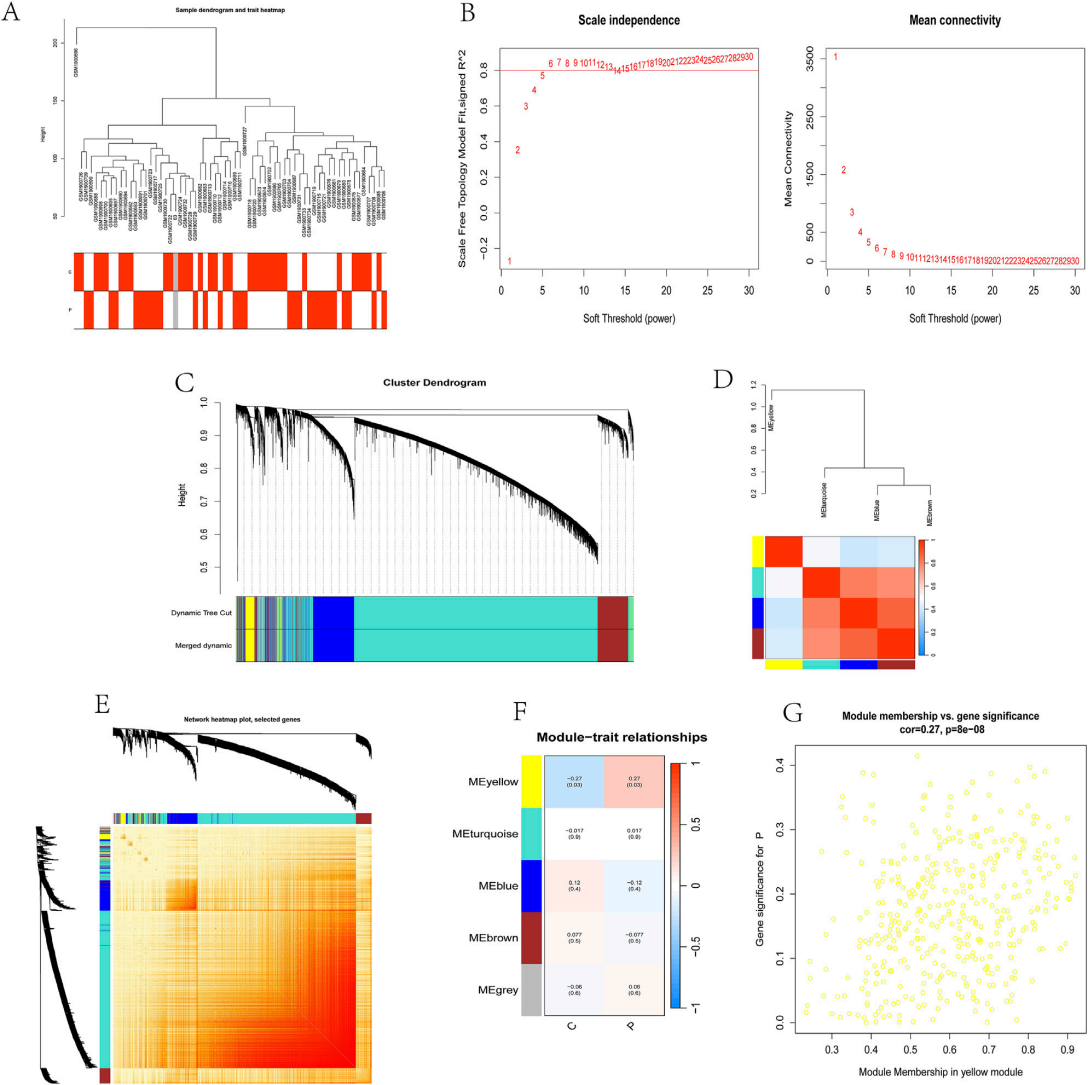
Selection of WGCNA modules and identification of hub genes.

A co-expression network was established through Weighted gene co-expression network analysis (WGCNA) with 32,077 genes, encompassing 33 control samples and 29 kidney stone samples. Initial steps involved sample clustering and outlier specimen exclusion using a predetermined threshold, as illustrated in Figure 2A. To ensure the network’s scale-free attribute and optimal average connectivity, a soft power threshold of 24 was set, resulting in a scale-free R^2 value of 0.9, as shown in Figure 2B.

Subsequently, under a clustering height cut-off of 0.25, we merged highly correlated modules and ultimately identified 5 modules for in-depth study. The initiation and merging processes of these modules were clearly displayed in the clustering tree (Figure 2C). To assess the correlation between these modules, we conducted correlation analysis, which revealed no significant associations between modules, demonstrating the rationality and independence of our module division (Figure 2D).

To validate the reliability of module characterization, we also performed intra-modular transcriptional correlation analysis, revealing no substantive relationships between modules, further confirming the accuracy of module division (Figure 2E). Additionally, we used module eigengenes (ME) to examine the correlation between module features and clinical traits, revealing a strong correlation between the yellow module and kidney stones (R = 0.27, P = 8e-08) (Figures 2F, G).

Based on this finding, we included 326 candidate genes from the yellow module in subsequent analyses. These genes may play important roles in the pathogenesis of kidney stones, providing new clues for our in-depth study of the biological mechanisms underlying kidney stones.

### 3.3 Functional enrichment analysis of overlapping DEGs

**FIGURE 3:**
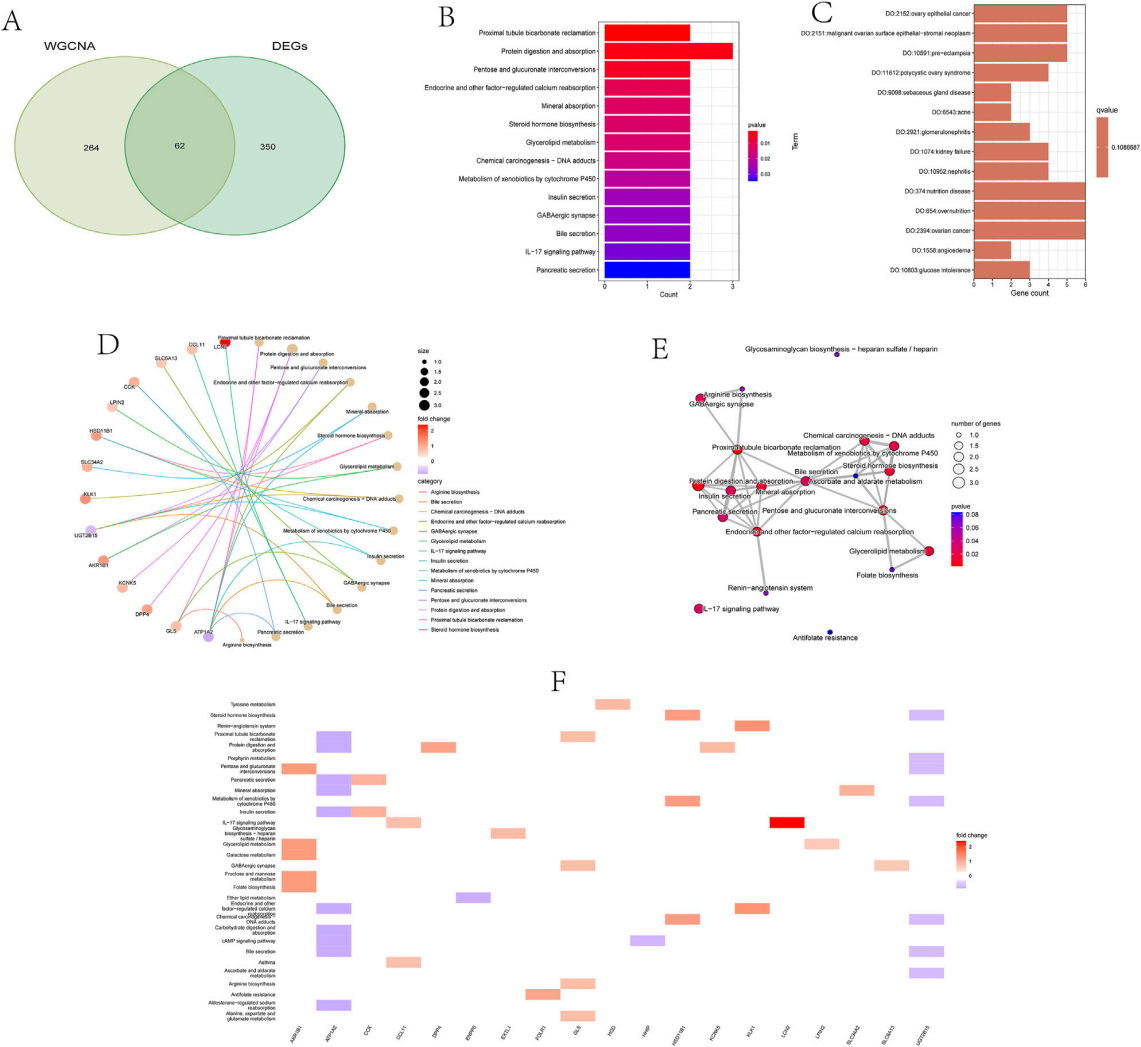
Functional enrichment analysis of overlapping differentially expressed genes (DEGs).

From the previously identified DEGs and genes in the yellow module, we successfully screened out 62 overlapping genes, named candidate feature genes, which were visually displayed in the figure (Figure 3A). To further elucidate the biological functions and enriched pathways that these candidate feature genes may be involved in, we performed KEGG (Kyoto Encyclopedia of Genes and Genomes) and DO (Disease Ontology) analyses.

KEGG enrichment analysis revealed signaling pathways that these candidate feature genes may participate in. These pathways include the proximal tubule bicarbonate reclamation pathway in the kidney, crucial for maintaining acid-base balance; protein digestion and absorption in the digestive system, providing amino acids to support energy production and tissue repair; pathways involving glucose and galactose metabolism in carbohydrate metabolism, critical for energy metabolism and biosynthesis processes; as well as multiple processes related to endocrine regulation, such as calcium reabsorption, mineral absorption, steroid hormone biosynthesis, and insulin secretion. Furthermore, these genes are also involved in chemical carcinogen metabolism, bile secretion, IL-17 signaling pathway, and other important biological processes (Figures 3B, D–F).

DO analysis showed that these candidate feature genes are mainly associated with various disease states. For example, they are significantly associated with metabolic abnormalities such as glucose intolerance and hyperglycemia, obesity, and nutrient excess states; as well as digestive system disorders such as constipation and intestinal dysmotility, and urinary system diseases such as renal failure and glomerulonephritis. Additionally, these genes are associated with immune system defects, cardiovascular diseases, various types of cancers, and other disease states such as arthritis, anemia, varicose veins, and connective tissue diseases. In summary, candidate feature genes play important roles in the pathogenesis of kidney stones (Figure 3C).

### 3.4 Identification of optimal feature genes through integration of multiple machine learning algorithms

**FIGURE 4:**
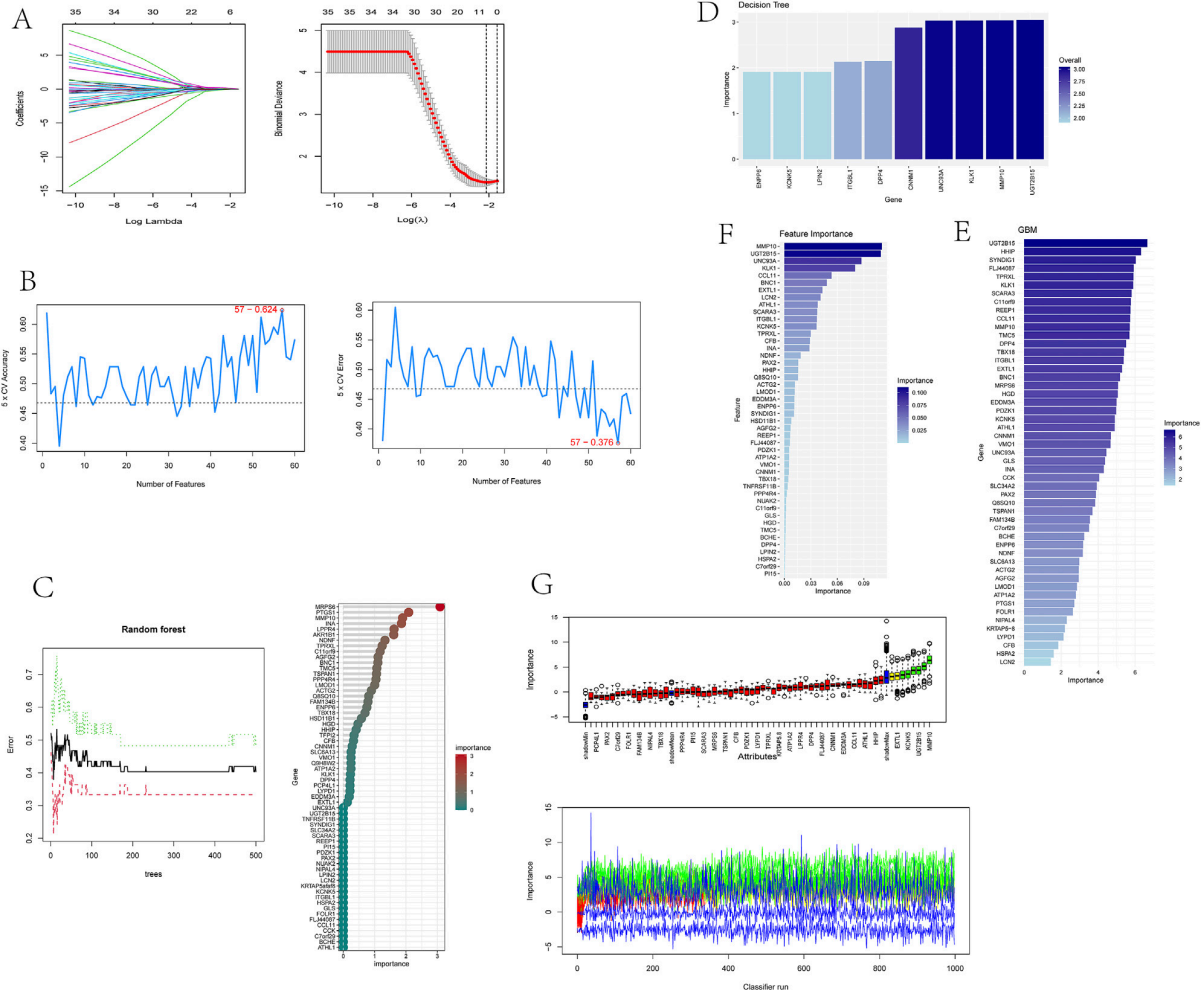
Identification of optimal feature genes through integration of multiple machine learning algorithms.

**FIGURE 5:**
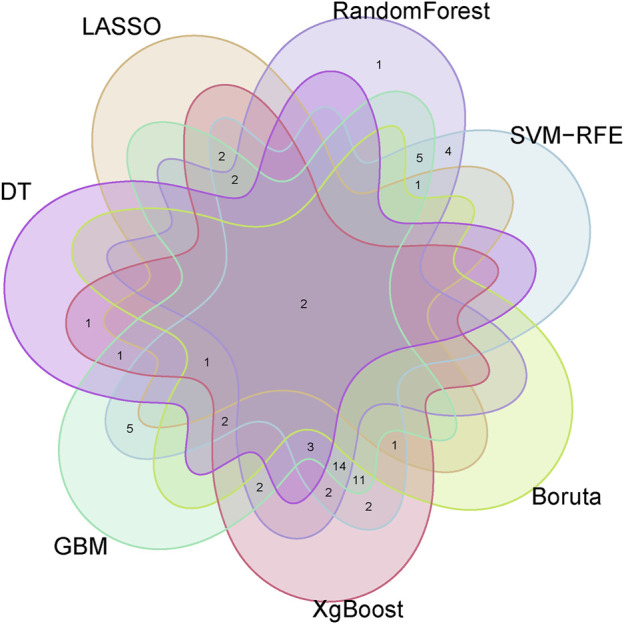
Venn diagram showing KLK1 and MMP10 as key feature genes identified across seven machine learning algorithms.

To identify putative feature genes, we employed seven different machine learning algorithms. Firstly, through LASSO analysis, we screened out 9 genes from the 62 candidate feature genes as diagnostic markers for kidney stones (Figure 4A). Simultaneously, we utilized the SVM-REF algorithm to cross-validate all candidate genes, demonstrating that all 62 genes have distinctive features (Figure 4B). Additionally, we used random forest (RF) algorithm to identify 36 feature genes with importance scores greater than 0 (Figure 4C), 10 key genes were identified through decision tree (DT) algorithm (Figure 4D), 51 feature genes were selected through gradient boosting machine (GBM) algorithm (Figure 4E), 46 important genes were determined using XgBOOST algorithm (Figure 4F), and 6 feature genes were confirmed by the Boruta algorithm (Figure 4G). Upon intersecting the feature genes identified by the seven aforementioned algorithms, two optimal feature genes, namely, KLK1 and MMP10, were successfully pinpointed (Figure 5). These two genes not only serve as potential diagnostic markers for kidney stones but also may be key genes in the progression of kidney stones.

### 3.5 Evaluation of expression and diagnostic significance of optimal feature genes

**FIGURE 6:**
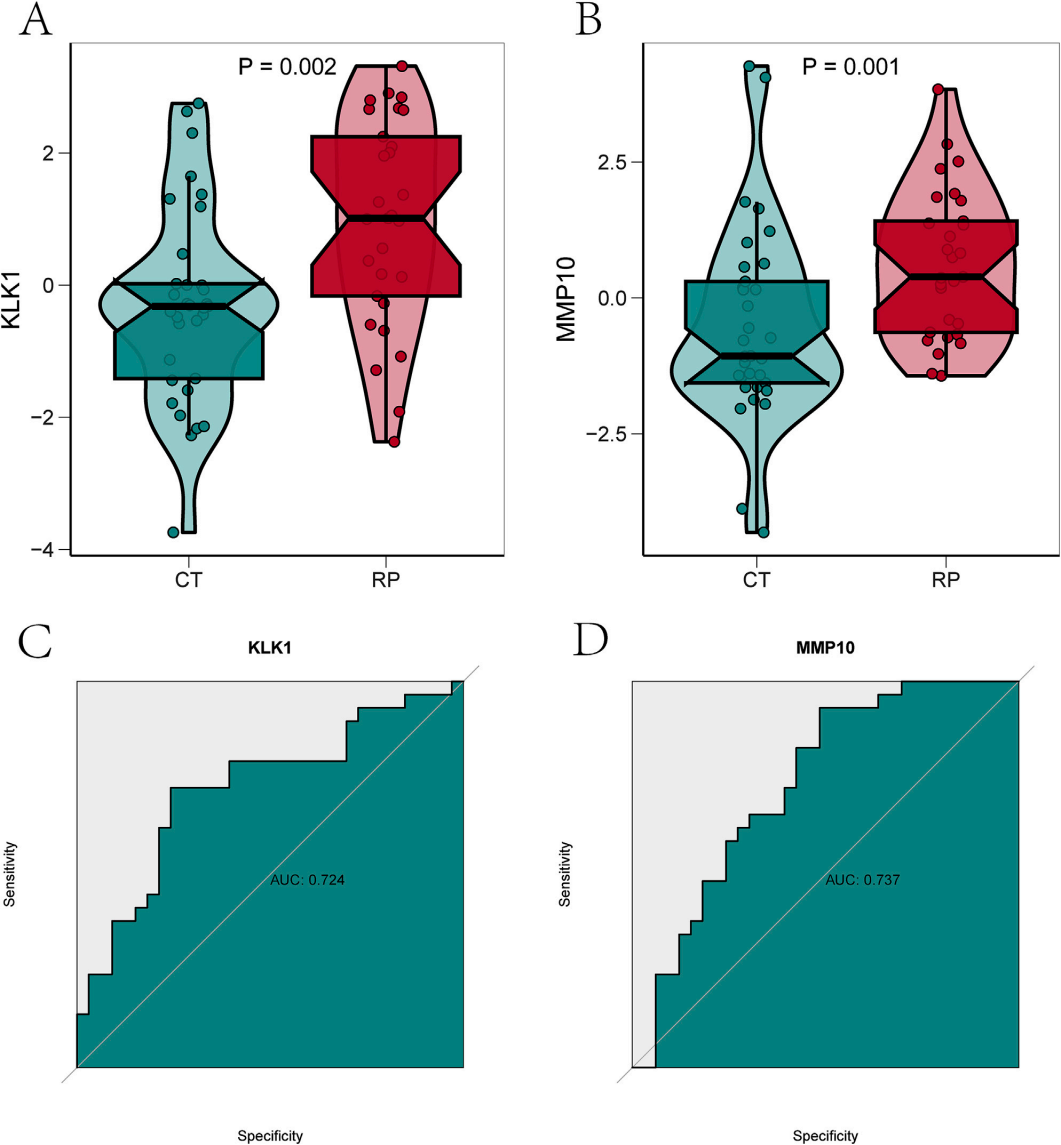
Evaluation of expression and diagnostic significance of optimal feature genes.

The expression levels of the two optimal feature genes, KLK1 and MMP10, were validated in 29 kidney stone samples and 33 normal samples. The results indicated a significant upregulation of KLK1 and MMP10 genes in kidney stone samples, suggesting their pivotal roles in kidney stone progression (*p* < 0.01) (Figures 6A, B). To further assess the diagnostic and predictive potential of these genes quantitatively, ROC curve analysis was conducted. The analysis revealed an AUC value of 0.724 for KLK1 and 0.737 for MMP10, highlighting their substantial diagnostic value in evaluating kidney stone progression (Figures 6C, D).

### 3.6 Functional identification of 2 feature genes

Given the important significance of these two feature genes in prognosis, we further conducted Gene Set Enrichment Analysis (GSEA) to explore their potential biological functions. We stratified kidney stone samples into two subgroups based on the median expression levels of these two optimal feature genes.

**FIGURE 7:**
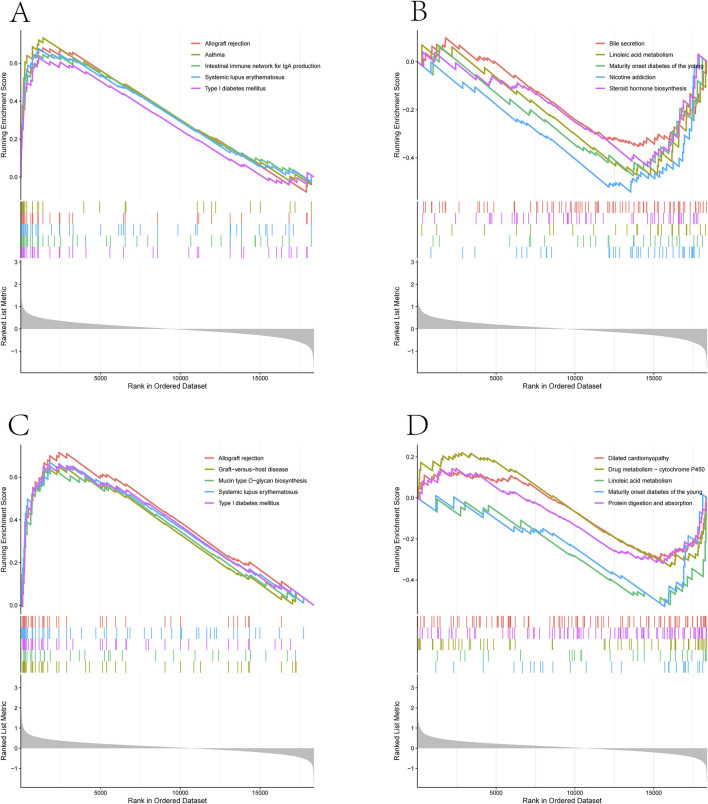
Functional identification of 2 feature genes.

For the high KLK1 expression subgroup, we found significantly enriched pathways related to asthma, allograft rejection, systemic lupus erythematosus, type I diabetes, and the impact on intestinal immune networks for IgA production, among others (Figure 7A). In contrast, in the low KLK1 expression subgroup, significantly enriched pathways included nicotine addiction, adolescent-onset type diabetes, linoleic acid metabolism, steroid hormone (Figure 7B).

In the high MMP10 expression subgroup, pathways significantly enriched included those associated with allograft rejection, systemic lupus erythematosus, type I diabetes, mucin-type O-glycan biosynthesis, and graft-versus-host disease (see relevant figures for details). Conversely, in the low MMP10 expression subgroup, pathways significantly enriched included adolescent-onset type diabetes, linoleic acid metabolism, drug metabolism-cytochrome P450, protein digestion and absorption, and dilated cardiomyopathy (Figure 7C).

It is noteworthy that, regardless of whether in the high expression group of KLK1 or MMP10, the type I diabetes pathway was significantly enriched. Whereas, in the low expression groups of KLK1 and MMP10, pathways related to adolescent-onset type diabetes and linoleic acid metabolism were significantly enriched (Figure 7D).

### 3.7 Signature gene sets and immune cell infiltration

**FIGURE 8:**
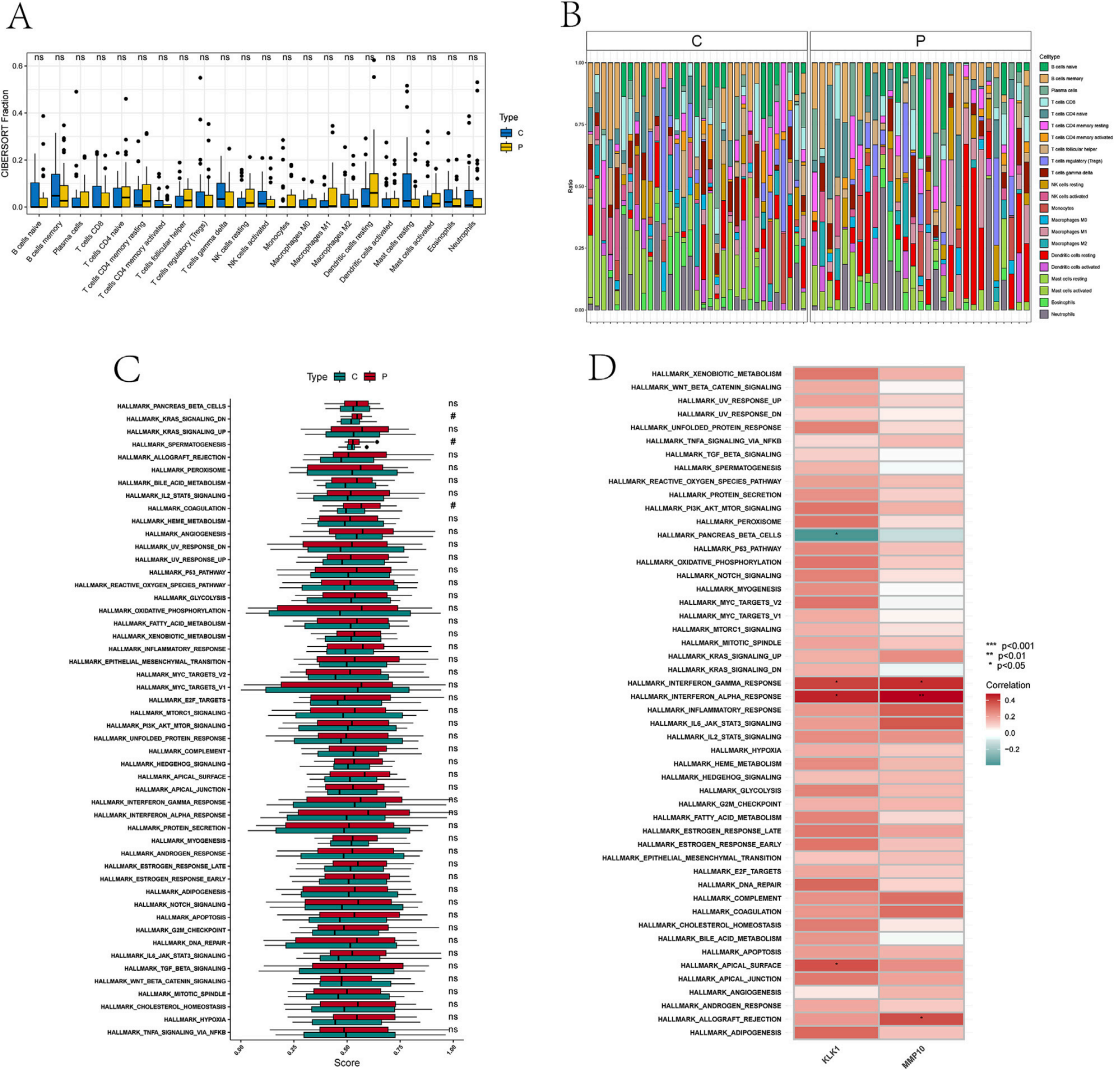
Signature gene sets and immune cell infiltration.

To assess variances in immune cell infiltration and signature gene sets between kidney stone patients and control samples, we utilized the CIBERSORT algorithm. Evaluation of differential immune cell infiltration revealed no substantial variances between the two groups at the immune cell level (Figures 8A, B). Furthermore, to explore enrichment variations in signature gene sets between the kidney stone and control groups, the ssGSEA algorithm was employed to analyze significant differences in 50 signature gene sets based on enrichment scores.

The analysis results revealed significant differences in the distribution of 50 signature gene sets between kidney stone patients and control samples. Specifically, several characteristic gene sets exhibited significant differences, including downregulation of typical KRAS signaling transduction, markers of spermatogenesis, and differences in the spermatogenesis gene set characteristics. Consequently, it can be reasonably inferred that these signature gene sets are overactivated in the kidney stone group compared to the normal group (Figure 8C).

Furthermore, we also found that the performance of the two optimal feature genes (KLK1 and MMP10) was generally consistent in most signature gene sets. For example, both of these feature genes showed positive correlations with the interferon gamma and alpha response signature gene sets. Furthermore, KLK1 was positively correlated with the apical surface signature gene set and negatively correlated with the pancreatic beta cell signature gene set, while MMP10 exhibited a positive correlation with the allograft rejection signature gene set (Figure 8D).

These findings provide important clues about the various roles that these two optimal feature genes may play in the pathogenesis of kidney stones, warranting further comprehensive investigation.

## 4 Discussion

In our study, we utilized a range of methods to comprehensively investigate the biological characteristics and potential mechanisms underlying kidney stones. Initially, we obtained the GSE73680 dataset from the GEO database, which includes gene expression profiles of normal renal papillary tissues and Randall’s plaques (RP) tissues. After rigorous screening using the R package “limma”, we identified 194 downregulated and 218 upregulated differentially expressed genes (DEGs). Subsequently, through gene set enrichment analysis using “ClusterProfiler”, we revealed key biological pathways closely associated with kidney stones. Additionally, we conducted weighted gene co-expression network analysis (WGCNA) to identify core functional modules during the pathogenesis of kidney stones.

In the subsequent integration of machine learning algorithms, we identified two optimal feature genes—KLK1 and MMP10, and confirmed their significant upregulation in kidney stone samples. Within proteases, renal KLK1 and matrix metalloproteinase-10 (MMP-10) have emerged as key players in renal function and blood pressure regulation (Sonkar et al., 2022; Golias et al., 2007). KLK1, initially discovered in human urine, is expressed in renal tubular epithelial cells and involved in electrolyte and water homeostasis, blood pressure regulation, and inflammation (Naicker et al., 1999). Studies have shown that renal KLK1 deficiency is associated with sodium retention and hypertension (Devetzi et al., 2018). MMP-10, also known as Stromelysin-2 or tansin-2, is a zinc-dependent endopeptidase (Wozniak et al., 2021; Tan and Liu, 2012). Although MMP-10 expression is barely detectable in development or normal adult tissues, it significantly increases after tissue injury (Zuo et al., 2021; McMahan et al., 2016; Koller et al., 2012; Lv et al., 2019). These findings not only provide new insights into the pathogenesis of kidney stones but also offer potential biomarkers for subsequent diagnostic and therapeutic research.

Given the potential importance of KLK1 and MMP10 in kidney stones, we further conducted gene set enrichment analysis (GSEA). The results showed significant enrichment of the type I diabetes pathway in both the high expression groups of KLK1 and MMP10, suggesting their association with the progression of diabetic nephropathy. Particularly in tubulointerstitial inflammation, KLK1 acts by activating PAR-4, providing a potential target for future diabetic nephropathy treatment (Yiu et al., 2014). The high expression of MMP-10 in chronic kidney disease (CKD), diabetes, and atherosclerosis also indicates its role as an important regulator of inflammation in these diseases (Coll et al., 2010; Toni et al., 2013; Mora-Gutiérrez et al., 2020). In the low expression groups of KLK1 and MMP10, pathways related to adolescent-onset type diabetes and linoleic acid metabolism were significantly enriched.

For a more precise evaluation of the diagnostic and predictive efficacy of these two genes in kidney stones, ROC curve analysis was performed. The results showed that the AUC (area under the curve) values of KLK1 and MMP10 were 0.724 and 0.737, respectively, both exceeding 0.7, indicating their high diagnostic value in evaluating the progression of kidney stones. This finding not only provides new insights into the pathogenesis of kidney stones but also offers potential biomarkers for future diagnostic and therapeutic strategies.

We conducted enrichment analysis of 50 signature gene sets using the ssGSEA algorithm, revealing significant differences in several signature gene sets between the kidney stone group and the control group, including downregulation of KRAS signaling transduction and markers of spermatogenesis. Particularly, KLK1 and MMP10 exhibited similar expression patterns in most signature gene sets, positively correlated with interferon gamma and alpha response, and associated with apical surface signature, pancreatic beta cell signature, and allograft rejection signature, suggesting their multifaceted roles in the pathogenesis of kidney stones.

The analysis highlighted several biases and limitations inherent in the employed machine learning algorithms. LASSO may exclude relevant features due to coefficient shrinkage and struggles with correlated variables. SVM-RFE’s sensitivity to kernel and regularization parameters, along with its computational demands, complicates its use with large datasets. Random Forest can overfit and shows bias towards features with more levels, while the Boruta algorithm may prioritize high-variance features, limiting its applicability due to computational constraints.

Ensemble methods like XGBOOST and GBM are prone to overfitting without careful hyperparameter tuning, complicating interpretability and making them sensitive to outliers. Decision Trees, although interpretable, are biased towards features with more levels and are prone to overfitting and instability. Data imbalance poses a risk of biased predictions across these models. The computational cost of ensemble methods impacts scalability, emphasizing the need for addressing these biases to ensure reliable and reproducible outcomes in future research.

The study’s findings have several clinical implications that could inform therapeutic strategies. By identifying algorithmic biases and limitations, clinicians and researchers can better select and fine-tune models to enhance predictive accuracy and interpretability. For instance, understanding LASSO’s tendency to exclude correlated features can guide the integration of complementary models to ensure comprehensive feature selection, crucial for personalized medicine approaches.

SVM-RFE and ensemble methods like Random Forest and XGBOOST, despite their challenges, offer robust frameworks for identifying key biomarkers, aiding in the development of targeted therapies. By addressing overfitting and interpretability issues, these models can be optimized for clinical decision-making, improving treatment efficacy and patient outcomes.

Moreover, recognizing the impact of data imbalance allows for the development of strategies to ensure fair and unbiased predictions, crucial for equitable healthcare delivery. Overall, the study underscores the importance of tailoring machine learning applications to clinical contexts, ultimately enhancing therapeutic interventions and advancing precision medicine.

In summary, our study not only provides new insights into the pathogenesis of kidney stones but also lays a solid foundation for future diagnostic and therapeutic research.

## 5 Limitations

First, limited sample size, the study’s findings are based on a relatively small sample size of 33 normal tissues and 29 RP tissues. This limitation may impact the generalizability and robustness of the results. In future research, we plan to incorporate a larger number of samples to enhance the reliability of our conclusions. Second, risk of Overfitting, due to the small dataset, there is a potential risk of overfitting, which could affect the accuracy and applicability of our findings. We aim to address this by employing more advanced statistical methods and incorporating a larger sample size in subsequent studies. Third, need for External Validation, our current study lacks validation with external datasets, which is crucial for confirming the diagnostic value of the markers identified. Future research will focus on validating our results using diverse external datasets to ensure the findings are applicable across different populations and settings.

## 6 Conclusion

Overall, our findings contribute to a better understanding of the molecular mechanisms underlying kidney stone formation and progression, laying a foundation for the development of novel diagnostic and therapeutic strategies. Further research is warranted to validate the clinical utility of identified biomarkers and explore their potential applications in personalized medicine for kidney stone patients.

## Data Availability

The raw data supporting the conclusions of this article will be made available by the authors, without undue reservation.
